# A Neurobiologically Constrained Cortex Model of Semantic Grounding With Spiking Neurons and Brain-Like Connectivity

**DOI:** 10.3389/fncom.2018.00088

**Published:** 2018-11-06

**Authors:** Rosario Tomasello, Max Garagnani, Thomas Wennekers, Friedemann Pulvermüller

**Affiliations:** ^1^Brain Language Laboratory, Department of Philosophy and Humanities, WE4, Freie Universität Berlin, Berlin, Germany; ^2^Centre for Robotics and Neural Systems, University of Plymouth, Plymouth, United Kingdom; ^3^Berlin School of Mind and Brain, Humboldt Universität zu Berlin, Berlin, Germany; ^4^Department of Computing, Goldsmiths, University of London, London, United Kingdom; ^5^Einstein Center for Neurosciences, Berlin, Germany

**Keywords:** word acquisition, semantic grounding, Hebbian learning, distributed neural assemblies, spiking neural network, brain-like connectivity

## Abstract

One of the most controversial debates in cognitive neuroscience concerns the cortical locus of semantic knowledge and processing in the human brain. Experimental data revealed the existence of various cortical regions relevant for meaning processing, ranging from semantic hubs generally involved in semantic processing to modality-preferential sensorimotor areas involved in the processing of specific conceptual categories. *Why* and *how* the brain uses such complex organization for conceptualization can be investigated using biologically constrained neurocomputational models. Here, we improve pre-existing neurocomputational models of semantics by incorporating spiking neurons and a rich connectivity structure between the model ‘areas’ to mimic important features of the underlying neural substrate. Semantic learning and symbol grounding in action and perception were simulated by associative learning between co-activated neuron populations in frontal, temporal and occipital areas. As a result of Hebbian learning of the correlation structure of symbol, perception and action information, distributed cell assembly circuits emerged across various cortices of the network. These semantic circuits showed category-specific topographical distributions, reaching into motor and visual areas for action- and visually-related words, respectively. All types of semantic circuits included large numbers of neurons in multimodal connector hub areas, which is explained by cortical connectivity structure and the resultant convergence of phonological and semantic information on these zones. Importantly, these semantic hub areas exhibited some category-specificity, which was less pronounced than that observed in primary and secondary modality-preferential cortices. The present neurocomputational model integrates seemingly divergent experimental results about conceptualization and explains both semantic hubs and category-specific areas as an emergent process causally determined by two major factors: neuroanatomical connectivity structure and correlated neuronal activation during language learning.

## Introduction

Although the brain mechanisms of meaning processing have been investigated for many years, cognitive neuroscientists have not reached a consensus about the function and the organizational principles of semantic knowledge. A range of neuroimaging and neuropsychological patient studies suggest a contribution of several cortical areas to semantic processing, but the precise role of each of them is still subject to debate. Cognitive and neuroscientists have suggested that the meanings of all words are equally processed and stored in a central “symbolic system” cortically located in a “semantic hub.” However, “semantic hubs” have been proposed in different cortical regions, including the anterior-inferior-temporal lobe (Patterson et al., 2007; Ralph et al., 2017), the anterior-inferior-parietal (Binder et al., 2009; Binder and Desai, 2011) and the posterior-inferior-frontal cortex (Posner and Pavese, 1998; Bookheimer, 2002; Tate et al., 2014; Schomers and Pulvermüller, 2016; Carota et al., 2017). Whereas it is possible, in principle, that several semantic hubs co-exist, some researchers postulated the need for bringing together all semantic information into one focal area and consequently reject the existence of multiple semantic hubs (Patterson et al., 2007; Ralph et al., 2017). Furthermore, and over and above semantic hubs generally contributing to all types of semantics, the phenomenon of category-specific semantic processing has long been in focus (McCarthy and Warrington, 1988; Shallice, 1988): modality-preferential cortices, including visual, auditory, olfactory, gustatory, somatosensory and motor regions, have been shown to differentially activate when specific semantic types are processed, for example animal vs. tool nouns or verbs typically used to speak about different types of actions (Damasio et al., 1996; Chao et al., 1999; Hauk et al., 2004; Kemmerer et al., 2012; Grisoni et al., 2016; Vukovic et al., 2017). Also studies of patients with lesions in modality-specific regions revealed category-specific semantic deficits (Warrington and Mccarthy, 1983; Damasio et al., 1996; Neininger and Pulvermüller, 2003; Gainotti, 2010; Trumpp et al., 2013; Dreyer et al., 2015) which can not be explained by symbolic systems accounts presuming category-general semantic hubs. Likewise, these findings challenge proposals that see the semantic processing role of sensorimotor areas as optional, ancillary or epiphenomenal and deny them a genuine semantic conceptual function (Machery, 2007; Mahon and Caramazza, 2008; Caramazza et al., 2014). The evidence for multiple hubs and modality-specific areas for conceptual-semantic knowledge is difficult to reconcile within most current neurobiological models of symbol processing.

To incorporate the diverging semantic theories and data from healthy and patient studies described above, it is necessary to build sophisticated models of relevant cortical areas that are biologically constrained by mimicking relevant features of brain function and connectivity. Ideally, such brain-constrained models may predict and offer mechanistic explanations for semantic processing in the human brain. Potentially, such modeling efforts can confirm a given theoretical framework, for example the existence of distributed semantic circuits spread out across several semantic hubs and modality-preferential areas or, as an alternative, the existence of a single focal “semantic hub.” Based on previous integrative proposals (Damasio, 1989; Pulvermüller, 2013), we hypothesize that semantic category-specific and category-general behaviors of different cortical areas are a direct consequence of the neuroanatomical connectivity between the areas involved and learning experiences that are essential for grounding concepts in knowledge about objects and actions. Here, we attempt to address this theoretical hypothesis with a neurobiologically constrained spiking model of the cortex in order to integrate data from healthy and patient studies described above.

Recent simulations of cortical function and learning incorporating fine microstructural and physiological details of millions of neurons (Izhikevich and Edelman, 2008; Markram et al., 2011) have not yet addressed specific questions about the neurobiological basis of specific cognitive functions such as semantic processing. Previous connectionist models have made significant progress in explaining of language and semantic processing (Dell et al., 1999; Plaut and Gonnerman, 2000; Christiansen and Chater, 2001), but most of them do not attempt to replicate realistic properties of the human brain. Although recent simulation studies included neuroanatomical information to model semantic processing, they have used learning mechanism (i.e., back-propagation—Ueno et al., 2011; Chen et al., 2017), which were argued to be biologically implausible (Mazzoni et al., 1991; O'Reilly, 1998). Furthermore, these studies have incorporated just one semantic hub area in the anterior temporal lobe, whereas other evidence summarized above are not addressed. A recent modeling effort incorporates neuroanatomical structure and connectivity into models of semantic processing (Garagnani and Pulvermüller, 2016). By meticulously mimicking the general parcellation of cortex into areas, their long-range cortico-cortical connections, features of local connectivity within cortical areas, local and global inhibitory mechanisms regulating cortical activity, and realistic neurobiological learning mechanisms, a stepwise approximation to response properties of real brain-internal networks could be achieved. Still, these previous study has fallen short of implementing the complexity of cortico-cortical connectivity and the activation dynamics of spiking cortical neurons.

Building upon these previous efforts with graded-response neural-network models (Garagnani and Pulvermüller, 2016), we here set out to model the brain's semantic mechanisms using a mathematically precise model of multiple cortical areas, incorporating spiking neurons, biologically plausible non-supervised learning mechanisms and connectivity structure based on neuroanatomical studies. The network was used to simulate associative word learning by linking word-forms with their semantically-related object and action representations. The present biologically constrained model bridges the gap between neural mechanisms and conceptual brain functions, offering a biological account of how aspects of word meaning are acquired, stored, and processed in the brain.

## Methods and materials

### General features of the model

**Figure 1:**
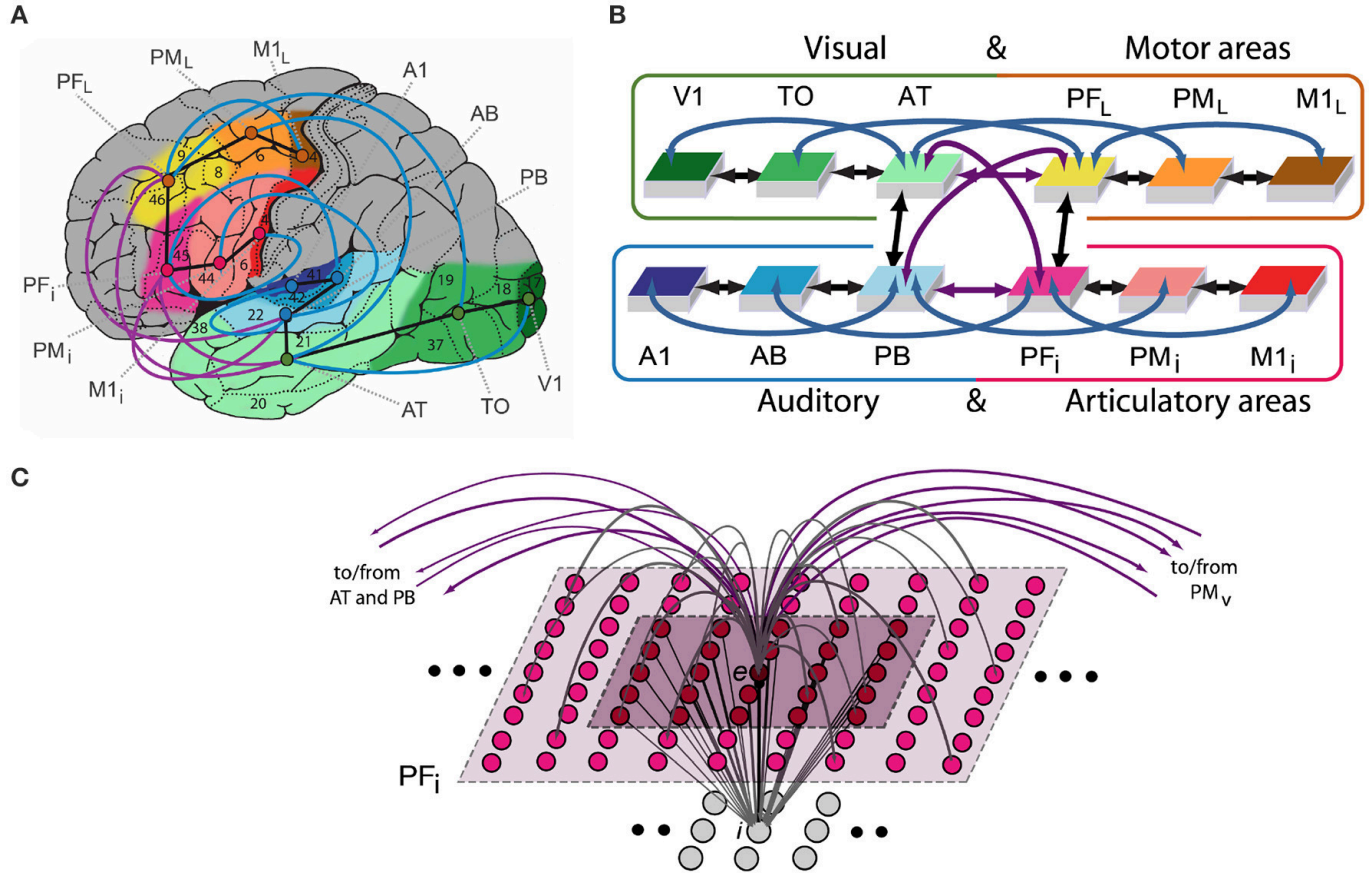
**(A)** Structure and connectivity of 12 frontal, temporal and occipital cortical areas relevant for learning the meaning of words related to actions. Perisylvian cortex comprises an inferior-frontal articulatory-phonological system (red colors), including primary motor cortex (M1_i_), premotor (PM_i_) and inferior-prefrontal (PF_i_), and a superior-temporal acoustic-phonological system (areas in blue), including auditory parabelt (PB), auditory belt (AB) and primary auditory cortex (A1). Extrasylvian areas comprise a lateral dorsal hand-motor system (yellow to brown), including lateral prefrontal (PF_L_), premotor (PM_L_) and primary motor cortex (M1_L_), and a visual “what” stream of object processing (green), including anterior-temporal (AT), temporo-occipital (TO), and early visual areas (V1). When learning words in the context of perceived objects or to actions, both peri- and extrasylvian systems are involved. Numbers indicate Brodmann Areas (BAs) and the arrows (black, purple, and blue) represent long distance cortico-cortical connections as documented by neuroanatomical studies. **(B)** Schematic global area and connectivity structure of the implemented model. The colors indicate correspondence between cortical and model areas. **(C)** Micro-connectivity structure of one of the 7,500 single excitatory neural elements modeled (labeled “*e*”). Within-area excitatory links (in gray) to and from cell *e* are limited to a local (19 × 19) neighborhood of neural elements (light-gray area). Lateral inhibition between *e* and neighboring excitatory elements is realized as follows: the underlying cell *i* inhibits *e* in proportion to the total excitatory input it receives from the 5 × 5 neighborhood (dark-purple shaded area); by means of analogous connections (not depicted), *e* inhibits all of its neighbors. Adapted from (Garagnani and Pulvermüller, 2013).

We implemented a neurobiologically constrained model replicating cortical areas of fronto-temporo-occipital lobes and their connectivity to shed light on the mechanism underlying semantic processing grounded in action and perception. We created a neural architecture with 15,000 representative neurons for simulating activity in twelve cortical areas in the left language-dominant hemisphere (see Figure 1A). These “areas” represented three levels of processing—primary, secondary, and higher-association cortex—in four modality-systems: (motor) frontal superior-lateral hand-motor, (articulatory) inferior face-motor, (auditory) superior-temporal and (visual) inferior-temporo-occipital system. Two of these, the auditory and articulatory systems (areas highlighted in blue and red, Figure 1A) are in perisylvian language cortex and appear most relevant for language processing (Zatorre et al., 1996; Pulvermüller, 1999; Fadiga et al., 2002; Pulvermüller and Fadiga, 2010). The motor and visual system (yellow and green highlighted areas) are outside the perisylvian language cortex (called “extrasylvian” in the present work) and involved in processing visual object processing (Ungerleider and Haxby, 1994), and the execution of manual actions (Deiber et al., 1991; Lu et al., 1994; Dum and Strick, 2002, 2005).

The model replicates a range of important anatomical and physiological features of the human brain (e.g., Garagnani et al., 2008, 2017; Tomasello et al., 2017). As follow a summary of the six neurobiological principles incorporated in the neural network model:

- Neurophysiological dynamics of spiking pyramidal cells including temporal summation of inputs, threshold-based spiking, nonlinear transformation of membrane potentials into neuronal outputs, and adaptation (Connors et al., 1982; Matthews, 2001);
- Synaptic modification by way of Hebbian-type learning, including the two biological mechanisms of long-term potentiation (LTP) and long-term depression (LTD) (Artola and Singer, 1993);
- Area-specific global regulation mechanisms and local lateral inhibition (global and local inhibition) (Braitenberg, 1978; Yuille and Geiger, 2003);
- Within-area connectivity: a sparse, random and initially weak connectivity was implemented locally, along with a neighborhood bias toward close-by links (Kaas, 1997; Braitenberg and Schüz, 1998);
- Between-area connectivity based on neurophysiological principles and motivated by neuroanatomical evidence; and
- Uncorrelated white noise was constant present in all neurons during all stages of learning and retrieval with additional noise added to the stimulus patterns to mimic uncorrelated input conditions (Rolls and Deco, 2010).

Note that the connectivity structure implemented in the network reflects existing anatomical pathways between corresponding cortical areas of the cortex revealed by neuroanatomical studies using diffusion tensor and diffusion-weighted imaging (DTI/DWI) in humans and non-human primates (Table 2) (Rilling et al., 2011; Thiebaut de Schotten et al., 2012). A detailed description of the single-neuron properties, synaptic plasticity rule, and single-area model structure is provided next, followed by details of the network anatomy and connectivity structure.

### Structure and function of the spiking model

Each of the 12 model areas consists of two layers of artificial neuron-like elements (“cells”), 625 excitatory and 625 inhibitory (*e*- and *i*-cells), thus resulting in 15,000 cells in total (see Figure 1C). Each *e*-cell models a single representative pyramidal spiking neuron situated in a local patch of the cortex and the underlying *i*-cell represents the cluster of inhibitory interneurons located within the same cortical column (Wilson and Cowan, 1972; Eggert and van Hemmen, 2000). The state of each cell *x* at time *t* is uniquely defined by its membrane potential *V*(*x,t*), specified by the following equation:

(B1) $$\begin{array}{l} \tau \cdot \frac{dV\left(x,t\right)}{dt}=-V\left(x,t\right)+k_{1}\left(V_{In}\left(x,t\right)+k_{2}\eta \left(x,t\right)\right) \end{array}$$

where *V*_*In*_ (*x,t*) is the net input acting upon cell *x* at time *t* (sum of all inhibitory and excitatory postsynaptic potentials—I/EPSPs; inhibitory synapses are given a negative sign), τ is the membrane's time constant, *k*_1_, *k*_2_ are scaling values (see Table 1 for the specific parameter values used in the simulations) and η(·,*t*) is a white noise process with uniform distribution over [−0.5, 0.5]. Note that noise is an inherent property of each model cell, intended to mimic the spontaneous activity (baseline firing) of real neurons. Therefore, noise was constantly present in all areas, in equal amounts (inhibitory cells have *k*_2_ = 0, i.e., the noise is generated by the excitatory cells). The output (or transformation function) φ of an excitatory cell *e* is defined as follows:

(B2) $$\begin{array}{l} \phi (e,t)=\{\begin{array}{ll} 1 & if\;(V(e,t)-\alpha \omega (e,t))>thresh\\ 0 & otherwise \end{array} \end{array}$$

Thus, an excitatory cell *e* spikes (=1) whenever its membrane potential *V*(*e,t*) overcomes a fixed threshold *thresh* by the quantity αω(*e,t*) (where α is a constant and ω is defined below). Inhibitory cells are graded response neurons, for simplicity, as they intend to represent the average impact of a cluster of local interneurons; the output φ(*i,t*) of an inhibitory neuron *i* is 0 if *V*(*i,t*) < 0 and *V*(*i,t*) otherwise.

**Table 1:** Parameter values used in the simulation.

Equation (B1)	Time constant (excitatory cells)	τ = 2.5 (simulation time-steps)
	Time constant (inhibitory cells)	τ = 5 (simulation time-steps)
	Total input rescaling factor	*k*_1_ = 0.01
	Noise amplitude	*k*_2_ = 5·√(24/Δt)
	Global inhibition strength	*k_*G*_* = 0.60
Equation (B2)	Spiking threshold	*Thresh* = 0.18
	Adaptation strength	α = 7.0
Equation (B3.1)	Adaptation time constant	*τ_*ADAPT*_* = 10 (time steps)
Equation (B3.2)	Rate-estimate time constant	*τ_*Favg*_* = 30 (time steps)
Equation (B3.3)	Global inhibition time constant	*τ_*GLOB*_* = 12 (time steps)
Equation (B4)	Postsynaptic membrane potential thresholds:	
		*ϑ_+_* = 0.15 *ϑ_−−_* = 0.14
	Presynaptic output activity required for LTP:	
		*ϑ_*pre*_* = 0.05
	Learning rate	Δ = 0.0008

To simulate neuronal adaptation (Kandel et al., 2000), the function ω(·*,t*) is defined so as to track the cell's most recent firing-rate activity. More precisely, the amount of adaptation ω(*e,t*) of cell *e* at time *t* is defined by:

(B3.1) $$\begin{array}{l} \tau_{ADAPT}\cdot \frac{d\omega \left(e,t\right)}{dt}=-\omega \left(e,t\right)+\phi \left(e,t\right) \end{array}$$

where τ_*ADAPT*_ is the “adaptation” time constant. The solution ω(*e,t*) of Equation (B3.1) is the low-pass-filtered output φ of cell *e*, which provides an estimate of the cell's most recent firing-rate history. A cell's average firing activity is also used to specify the network's Hebbian plasticity rule [see Equation (B4) below]; in this context, the (estimated) instantaneous mean firing rate ω_*E*_(*e*,*t*) of an excitatory neuron *e* is defined as:

(B3.2) $$\begin{array}{l} \tau_{Favg}\cdot \frac{d\omega_{E}\left(e,t\right)}{dt}=-\omega_{E}\left(e,t\right)+\phi \left(e,t\right) \end{array}$$

To regulate and control activity in the network, local and area-specific inhibition is implemented (Palm, 1982; Bibbig et al., 1995; Wennekers et al., 2006), realizing, respectively, local and global competition mechanisms (Duncan, 1996, 2006). More precisely, in Equation (B1) the input *V*_*In*_(*e,t*) to each excitatory cell of the same area includes an area-specific (“global”) inhibition term *k*_*G*_ω_*G*_(*e*,*t*) [with *k*_*G*_ a constant and ω_*G*_(*e*,*t*) defined below] subtracted from the total I/EPSPs postsynaptic potentials *V*_*In*_ in input to the cell; this regulatory mechanism ensures that area (and network) activity is maintained within physiological levels (Braitenberg and Schüz, 1998):

(B3.3) $$\begin{array}{l} \tau_{GLOB}\cdot \frac{d\omega_{G}\left(e,t\right)}{dt}=-\omega_{G}\left(e,t\right)+\underset{e\in area}{\sum }\varphi \left(e,t\right) \end{array}$$

Excitatory links within and between (possibly non-adjacent) model areas are established at random and limited to a local (topographic) neighborhood; weights are initialized at random, in the range [0, 0.1]. The probability of a synapse to be created between any two cells falls off with their distance (Braitenberg and Schüz, 1998) according to a Gaussian function clipped to 0 outside the chosen neighborhood (a square of size *n* = 19 for excitatory and *n* = 5 for inhibitory cell projections). This produces sparse, patchy and topographic connectivity, as typically found in the mammalian cortex (Amir et al., 1993; Kaas, 1997; Braitenberg and Schüz, 1998; Douglas and Martin, 2004).

The Hebbian learning mechanism implemented simulates well-documented synaptic plasticity phenomena of long-term potentiation (LTP) and depression (LTD), as implemented by Artola, Bröcher and Singer (Artola et al., 1990; Artola and Singer, 1993). This rule provides a realistic approximation of known experience-dependent neuronal plasticity and learning (Musso et al., 1999; Rioult-Pedotti et al., 2000; Malenka and Bear, 2004; Finnie and Nader, 2012), and includes both (homo- and hetero-synaptic, or associative) LTP, as well as homo- and hetero-synaptic LTD. In the model, we discretized the continuous range of possible synaptic efficacy changes into two possible levels, +Δ and –Δ (with Δ < < 1 and fixed). Following Artola et al., we defined as “active” any (axonal) projection of excitatory cell *e* such that the estimated firing rate ω_*E*_(*e*,*t*) of cell *e* at time *t* [see Equation (B3.2)] is above ϑ_*pre*_, where ϑ_*pre*_ ∈ ]0,1] is an arbitrary threshold representing the minimum level of presynaptic activity required for LTP (or homosynaptic LTD) to occur. Thus, given a pre-synaptic cell *i* making contact onto a post-synaptic cell *j*, the change Δ*w*(*i,j*) inefficacy of the (excitatory-to-excitatory) link from *i* to *j* is calculated as follows:

(B4) $$\begin{array}{l} \Delta w(i,j)=\{\begin{array}{lll} +\Delta & if\omega_{E}(i,t)\geq \vartheta_{pre}\;andV(j,t)\geq \vartheta_{+} & \left(LTP\right)\\ -\Delta & if\omega_{E}(i,t)\geq \vartheta_{pre}and\;\vartheta_{-}\leq V(y,t)<\vartheta_{+} & \left(homosynapticLTD\right)\\ -\Delta & if\omega_{E}(i,t)<\vartheta_{pre}andV(y,t)\geq \vartheta_{+} & \left(heterosynapticLTD\right)\\ 0 & otherwise & \; \end{array} \end{array}$$

The values in Table 1 describes the parameters used during word learning simulation in the network, which were chosen on the basis of previous simulations (e.g., Garagnani et al., 2007, 2009; Garagnani and Pulvermüller, 2011; Schomers et al., 2017; Tomasello et al., 2017).

### Simulated brain areas and their connectivity structure

The spiking model mimics 12 different cortical areas with area-intrinsic connections and mutual connections between them. Six areas were modeled for the left-perisylvian language cortex including the primary auditory cortex (A1), auditory belt (AB), and modality-general parabelt areas (PB) constituting the auditory system, and the inferior part of primary motor cortex (M1_i_), inferior premotor (PM_i_) and multimodal prefrontal motor cortex (PF_i_) representing the articulatory system (i.e., inferior face-motor areas). Additionally, six extrasylvian areas were modeled including the primary visual cortex (V1), temporo-occipital (TO) and anterior-temporal areas (AT) for the ventral visual system and the dorsolateral fronto-central motor (M1_L_), premotor (PM_L_), and prefrontal cortices (PF_L_) for the motor system.

The network's connectivity structure reflects relevant features of cortical connectivity between corresponding areas of the cortex. These were modeled between neighbor cortical areas within each of the 4 “streams” (see black arrows Figures 1A,B) and between all pairs of multimodal areas (PB, PF_i_, AT, and PF_L_) through the long distance cortico-cortical connections (purple arrows). Additionally, non-adjacent “jumping” links were included within the superior or inferior temporal and superior or inferior frontal cortices (blue arrows). The neuroanatomical evidence motivated by studies using diffusion tensor and diffusion-weighted imaging (DTI/DWI) in humans and non-humans primates are reported in Table 2 and described in previous study (Garagnani et al., 2017).

**Table 2:** Connectivity structure of the modeled cortical areas.

**Between-Area Connectivity (black arrows)**	
**Modeled areas**	**References**
**Perisylvian System**	
A1, AB, PB	Pandya, 1995; Kaas and Hackett, 2000; Rauschecker and Tian, 2000
PF_i_, PM_i_, M1_i_	Pandya and Yeterian, 1985; Young et al., 1995
**Extrasylvian System**	
V1, TO, AT	Bressler et al., 1993; Distler et al., 1993
PF_L_, PM_L_, M1_L_	Pandya and Yeterian, 1985; Arikuni et al., 1988; Lu et al., 1994; Rizzolatti and Luppino, 2001; Dum and Strick, 2002, 2005
**Between System**	
AT, PB	Gierhan, 2013
PF_i_, PF_L_	Yeterian et al., 2012
**LONG DISTANCE CORTICO-CORTICAL CONNECTIONS (PURPLE ARROWS)**	
**Perisylvian System**	
PF_i_, PB	Meyer et al., 1999; Romanski et al., 1999b; Paus et al., 2001; Catani et al., 2005; Parker et al., 2005; Rilling et al., 2008; Makris and Pandya, 2009
**Extrasylvian System**	
AT, PF_L_	Bauer and Jones, 1976; Fuster et al., 1985; Ungerleider et al., 1989; Eacott and Gaffan, 1992; Webster et al., 1994; Parker, 1998; Chafee and Goldman-Rakic, 2000
**Between System**	
PB, PF_L_	Pandya and Barnes, 1987; Romanski et al., 1999a,b
AT, PF_i_	Pandya and Barnes, 1987; Ungerleider et al., 1989; Webster et al., 1994; Romanski, 2007; Petrides and Pandya, 2009; Rilling, 2014
**HIGH-ORDER “JUMPING” LINKS (BLUE ARROWS)**	
**Perisylvian System (Rilling et al., 2008, 2011; Thiebaut de Schotten et al., 2012; Rilling and van den Heuvel, 2018)**	
A1, PB	Pandya and Yeterian, 1985; Young et al., 1994
PB, PMi	Rilling et al., 2008; Saur et al., 2008
AB, PFi	Romanski et al., 1999a; Kaas and Hackett, 2000; Petrides and Pandya, 2009; Rauschecker and Scott, 2009
PFi, M1i	Deacon, 1992; Young et al., 1995; Guye et al., 2003
**Extrasylvian System (see also Thiebaut de Schotten et al., 2012)**	
V1, AT	Catani et al., 2003; Wakana et al., 2004
AT, PML	Bauer and Fuster, 1978; Fuster et al., 1985; Pandya and Barnes, 1987; Seltzer and Pandya, 1989; Chafee and Goldman-Rakic, 2000
TO, PF_L_	Bauer and Jones, 1976; Fuster and Jervey, 1981; Fuster et al., 1985; Seltzer and Pandya, 1989; Makris and Pandya, 2009
PF_L_, M1_L_	Deacon, 1992; Young et al., 1995; Guye et al., 2003

### Simulating word acquisition

**Figure 2:**
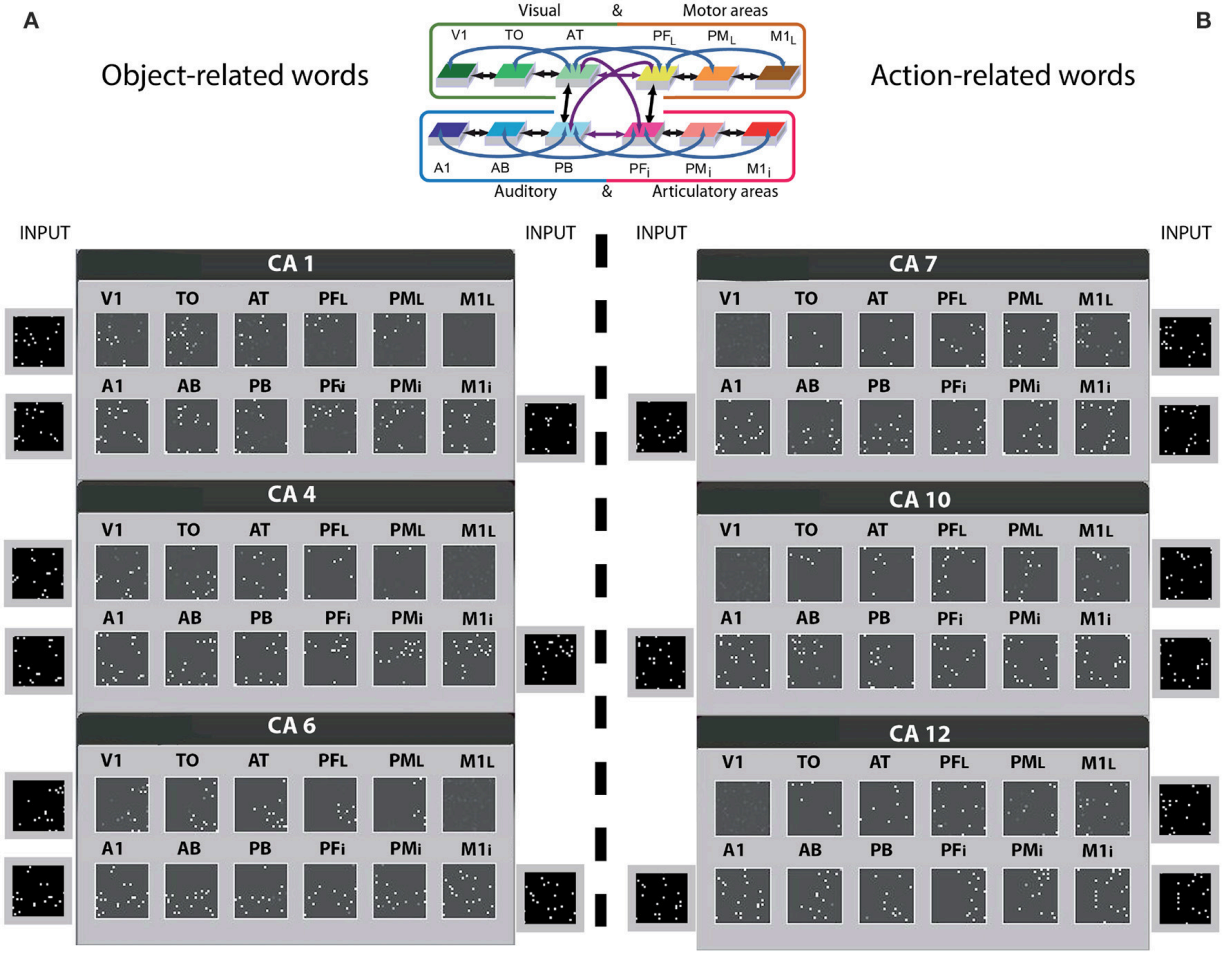
Distributions of cell-assemblies (CAs) emerging in the 12 area network during simulation of word learning in the semantic context of visual perception **(A)** and action execution **(B)**. Results of one typical instantiation of the model in Figure 1B are shown, using the same area labels. Each set of 12 squares (in black) illustrates one specific network area, with white dots indexing the distribution of CA neurons across the 12 network areas as a result of sensorimotor pattern presentation in 3 of the 4 primary areas. The perisylvian cortex was always stimulated, which mimics the learning of a spoken word form characterized by articulatory-acoustic features, while object words **(A)** received concordant stimulation to visual area (V1) and action words **(B)** to motor area (M1_i_). Note that a random pattern simulating realistic noise input, changing in every learning phase, was presented to the non-relevant system (see Methods section). As a consequence of learning, CA circuits emerged in the network which extends into higher and primary visual cortex (V1, TO, but not M1_L_) for object words. In contrast, network correlates of action-related words extend into lateral motor cortex (M1_L_, PM_L_, but not V1), thus semantically grounding words in information about actions. For convenience, the area structure of the network is repeated at the top.

Prior to network training, all synaptic links (between- and within-areas) connecting single cells were established at random (see Methods section under “*Structure and function of the spiking model”*). Based on Hebbian (Hebb, 1949) learning principles, word-meaning acquisition was simulated under the impact of repeated sensorimotor pattern presentations (Fuster, 2003; D'Esposito, 2007) to the primary areas of the network (see Figure 2), as follows: Each network instance used twelve distinct sets of sensorimotor neural patterns representing six action- and six object-related words. Each pattern consisted of a fixed set of 19 cells chosen at random within the 25 × 25 cells of an area (ca. 3% of the cells) and simultaneously activated in one of the primary areas of the network. The learning of object- and action-related words were grounded in sensorimotor information presented to the primary cortices of the model: besides perisylvian auditory A1 and articulatory M1_i_ activity, object-related words received concordant visual (V1) and, similarly, action-related words received lateral motor area (M1_L_) grounding activity. Note that white (so-called “contextual”) noise was continuously presented to all primary areas of the network, and thus superimposed on all learning patterns. This partly accounted for the variability of perceptions and actions of the same type. To sum up, the network was set up to learn correlations between word and referential semantic information in action and perception and to investigate which type of representations (i.e., cell assemblies) would develop in the model as a result of learning and cortical structure. Note that similar approaches to simulating spontaneous emergence of associations between articulatory and acoustic-phonetic neural patterns have been used in other computational studies (e.g., Westermann and Reck Miranda, 2004; Guenther et al., 2006), although these previous works did not attempt to model semantic processes (i.e., word meaning acquisition).

Sensorimotor neural patterns in the arrangement of 3 × 19 cells, were presented for 3,000 times to the relevant primary regions (this number was chosen on the basis of previous simulations obtained with a six area model, showing that no substantial change between 1,000 and 2,000 learning steps was revealed, Garagnani et al., 2009; Schomers et al., 2017). A word pattern was presented for 16 simulation time steps, followed by a period during which no input (interstimulus interval—ISI) was given. The next learning step (pattern presentation) occurred only when the global inhibition of PF_i_ and PB areas reduced below a specific fixed threshold allowing the activity to return to a baseline value so that one trial is not affecting the next one. Only the inherent baseline noise (simulating spontaneous neuronal firing) and “contextual” noise were present in the neural network during each ISI.

After learning, following a procedure which has become standard in our simulation studies (Garagnani et al., 2008; Garagnani and Pulvermüller, 2016; Schomers et al., 2017; Tomasello et al., 2017), we identified and quantified the neurons forming the 12 distributed CA circuits that emerged across the network areas during object and action word production. For simulating “word production” in the network, the motor and auditory neurons of each word form in areas M1 and A1 were activated together for 15 time-steps. Separate analyses were performed for object recognition and action execution, which was simulated by activating the corresponding stimulation pattern in visual or motor cortex (V1 or M1) thought to represent the object-related or action-related schemas semantically linked to the word forms. During this period, we computed and displayed the average firing rate of each excitatory cell (7,500 *e*-cells, cell's responses).

As an estimate of a cell's average firing-rate here we used the value ω_*E*_(*e*,*t*) from Equation (B3.2), integrated with time-constant τ_*Favg*_ = 5. An *e*-cell was then taken to be a member of a given CA circuit only if its time-averaged rate (output value or “firing rate”) reached a threshold θ which was area- and cell-assembly specific, and defined as a fraction γ of the maximal single-cell's time-averaged response in that area to pattern *w*. More formally,

$$\theta =\theta_{\text{A}}\left(w\right)=\gamma \;\underset{x\in A}{max}\bar{O{\left(x,t\right)}_{w}}$$

where $\bar{O{\left(x,t\right)}_{w}}$ is the estimated time-averaged response of cell *x* to word pattern *w* (see in Method section under “*Structure and function of the spiking model*”) and γ ∈ [0, 1] is a constant [we used γ = 0.5 on the basis of previous simulation results (see Garagnani et al., 2008, 2009; Tomasello et al., 2017)]. This was computed for each of the 12 trained network instances, averaging the number of CA cells per area over the 6 object- and 6 action-related words.

To statistically test for the presence of significant differences in the topographical CA distribution across the twelve network areas, for each network instance we performed a repeated-measures Analyses of Variance (ANOVA). A 4-way ANOVA was run with factors WordType (two levels: *Object* vs. *Action*), PeriExtra (two levels: *Perisylvian* = {A1, AB, PB, M1_i_, PM_i_, PF_i_}, *Extrasylvian* cortex = {V1, TO, AT, M1_L_, PM_L_, PF_L_}), TemporalFrontal (TempFront)” (2 levels: *temporal areas* = {A1, AB, PB, V1, TO, AT}, *frontal areas* = {M1_L_, PM_L_, PF_L_, M1_i_, PM_i_, PF_i_}) and Areas (three levels: *Primary* = {A1, V1, M1_L_, M1_i_}, *Secondary* = {TO, AB, PM_L_, PM_i_} and *Central* = {PB, AT, PF_L_, PF_i_} areas). Finally, we further run a second statistical analysis on the data of the 6 perisylvian and 6 extrasylvian areas separately with factors “WordType,” “TempFront,” “Areas,” as described above.

## Results

### Word learning results

Twelve different instances of spiking networks were initialized at random having the same architecture as described above (Figure 1B), providing analogs of 12 human subjects in a word learning experiment. Word-meaning acquisition was then simulated under the impact of repeated sensorimotor pattern presentations, in the 3 of the 4 sub-systems (see Figure 2), by co-activating specific neurons in their respective primary cortex. The cells activated in M1_i_ and A1 represented articulatory and acoustic-phonetic features by which spoken words are typically characterized, while those presented to V1 and M1_L_ simulated visually-related and action-related semantic features. This simulates associative learning of object-related word, whereby the word is uttered while the referent object is present (Vouloumanos and Werker, 2009) or the related action is being performed (Tomasello and Kruger, 1992). While each learning pattern directly activated three primary areas, the fourth unrelated area (M1_i_ for object- and V1 for action-related words) received further uncorrelated noise pattern input that changed inconsistently over learning episodes. This aimed at ensuring that the correlation between word-form activity in perisylvian cortex and semantic information was high in one modality (for action /object words, in motor and visual systems respectively) but low in the non-relevant one.

Cell assemblies gradually emerged as a consequence of learning with different assemblies responding to different input patterns. These neural circuits spanned different areas, linking up word-forms in the auditory and articulatory sub-systems with referential-semantic information in the visual and motor sub-systems. Figure 2 illustrates 6 of the 12 CA-distributions emerging across the novel spiking network along with the sensorimotor pattern presented as input during learning. Each set of 12 squares is a snapshot of a distributed word-related CA circuit across the network areas; 3 for object-related words (A) and 3 for action-related (B) words of one network instance (the other simulated networks exhibited similar results). Each white pixel in the squares represents an active cell of the CA.

The CA circuits in Figure 2 show roughly the same spread across the perisylvian areas for object and action-related words. By contrast, the visual and motor sub-systems of the extrasylvian cortex appear to show a different pattern of CA cell distribution, namely a double dissociation, i.e., object-related words seemed to extend more to the visual areas (V1, TO) and less to the motor areas (PM_L_, M1_L_) and vice versa for action-related words.

**Figure 3:**
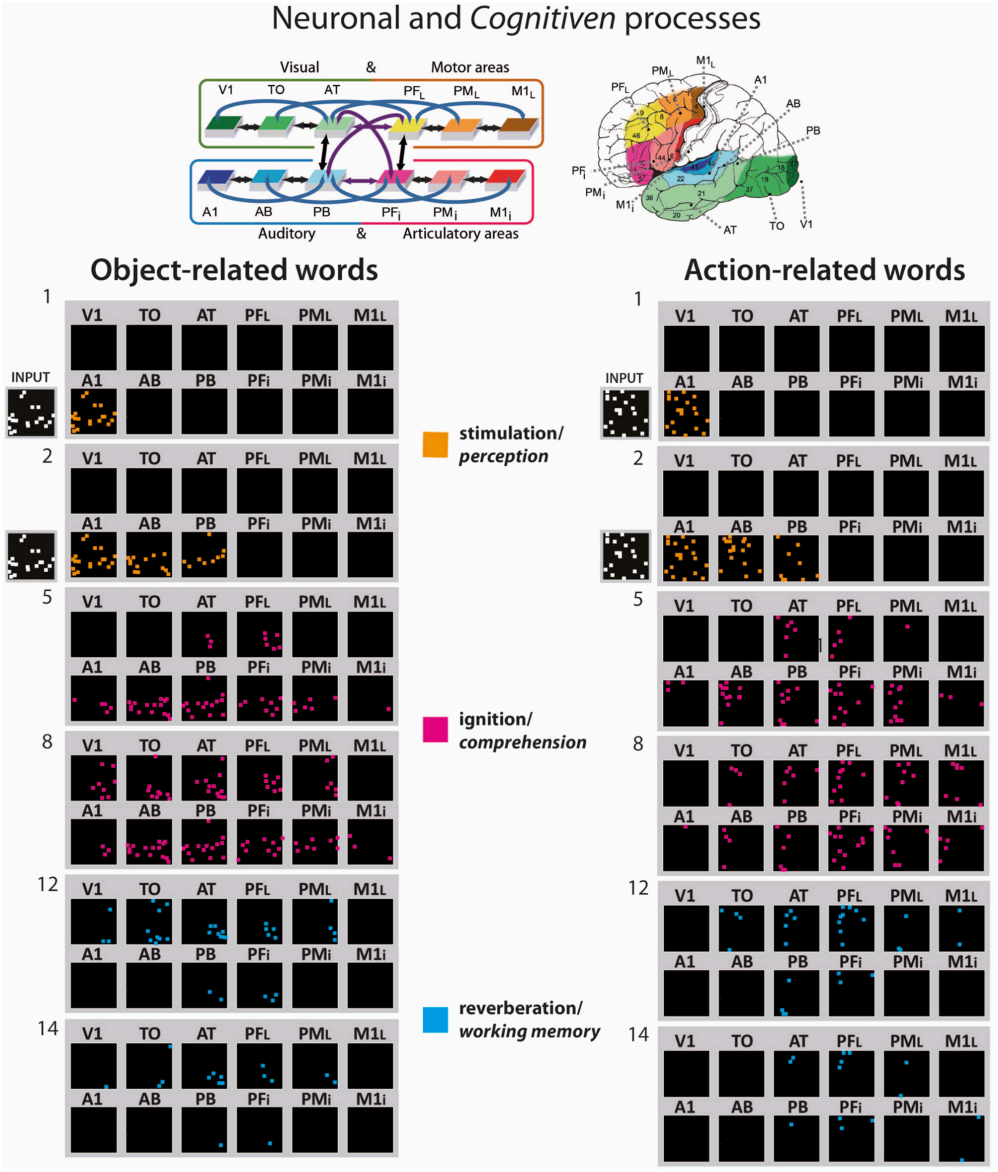
Activation spreading in the 12 area network showing examples of the simulated recognition processes for object- and action-related words (on the left and right, respectively; see CA #6 and CA #10 in Figure 2, respectively). Network responses to stimulation of A1 with the “auditory” patterns of two of the learned words; similar to Figure 2, the 12 network areas are represented as 12 squares, but, in this case, selected snapshots of network activity are shown. The re-activation process comes in different consecutive neuronal and cognitive phases, the stimulation phase, which corresponds to word perception (orange pixel), the full activation or “ignition” phase, the correlate of word comprehension (magenta pixel), and the reverberant maintenance of activity, which underpins verbal working memory (blue pixels). Each colored pixel indicates one spike one neuron included in the CA circuit at a given time step. At the top, the 12 model areas and their connectivity structure are shown and their location in the cortex indicated.

Figure 3 illustrates examples of CA circuit activation (i.e., each white pixel represents a spike) after the training has been undertaken. The network was confronted with the acoustic component (input pattern in primary auditory area) representing the auditory word-forms of the learned (A) object- and action-related (B) words, which in turn caused the “ignition” of the whole CA circuit for that specific word-pattern. The snapshot numbers indicate simulation time-steps of the network activity. Similarly, as in the distribution of the emerging CA circuits illustrated in Figure 2, action- and object-related word recognition exhibited a semantic category-specific spreading of activity in the modality-preferential areas, which is near simultaneous (i.e., synchronous spikes) binding information from phonological (articulatory-acoustic) and semantic information. Interestingly, the re-activation of the word-related cell assemblies across the cortical areas exhibit the distinct consecutive neuronal and cognitive processes; the stimulation phase (time steps 1–2), which corresponds to word perception (orange pixel), the full activation or “ignition” phase (time steps 5–8), the correlate of word comprehension (magenta pixel), and the reverberant maintenance of activity (time steps 12–14), which underpins verbal working memory (blue pixels).

**Figure 4:**
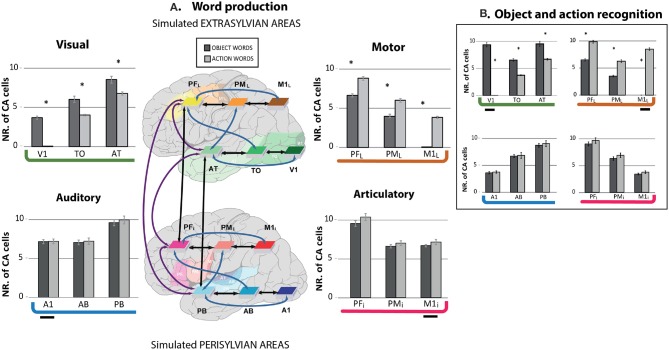
Mean numbers of cell assembly neurons in different model areas after simulating the learning of action- (light gray) and object-related words (dark gray) during word production **(A)** and object and action recognition **(B)**; error bars show standard errors over networks. **(A)** Simulated word production (simultaneous presentation of articulatory-auditory patterns in A1 and M1i areas) after word meaning acquisition. The extrasylvian areas (upper part) whose cells can be seen as circuit correlates of word meaning show a double dissociation, with relatively more strongly developed CAs for object- than for action-related words in primary and secondary visual areas (V1, TO), but stronger CAs for action-related than for object-related words in dorsolateral primary motor and pre-motor cortices (PM_L_, M1_L_). Also, the semantic hub areas (PFi, AT) showed a degree of dissociation between the two word types. Data from the perisylvian cortex (lower part), namely articulatory and auditory areas, whose cells can be seen as circuit correlates of spoken word-forms do not show category-specific effects. Brain areas and their connectivity structure are also illustrated. The shaded areas, but not the colored boxes, indicate location in the cortex. **(B)** Simulated object and action recognition [alternated presentation of sensorimotor patterns in visual (for object) and in motor areas (for action words)]. The present simulation exhibits similar results to the word production simulation. The small horizontal segment indicates the stimulus input presentation. Asterisks indicate that, within a given area, the number of CA cells significantly differed between the circuits of action and object words (Bonferroni-corrected planned comparison tests).

The bar graph in Figure 4 reports the topographical distribution of the CA circuits across the network areas averaged over 12 networks. Different panels show results from the word production (A) and object and action recognition (B) “experiments.” In each panel, average numbers of cell assembly neurons (plus standard errors) are shown for each area, with extrasylvian areas displayed at the top and perisylvian ones at the bottom. Intriguingly, the extrasylvian areas show a different CA distribution between the two word-type circuits, while the perisylvian language areas seem not to show any word-category differences.

Furthermore, independently of whether an object or action-related word is represented, the word learning results showed higher density of CA cells in the connector hubs (PB, PF_i_, AT, and PF_L_) than in the secondary (AB, PM_i_, TO, PM_L_) and primary areas (A1, M1_i_, V1, M1_L_). Similar results were revealed for both word production and action and object recognition, which is in line with the differential CA topographies already noted above and in Figure 2. However, there were minor differences in the estimated cell assembly topographies, as the relatively larger number of CA cells in the primary areas of the extrasylvian system were obtained for object and action recognition compared to word production, which was (trivially) due to the stimulus presentation there.

The 4-way repeated measurement ANOVA (with factors WordType, PeriExtra, TemporalFrontal, and Areas) performed on the word production data from all of the 12 network areas fully confirmed the empirical and visual observation described above. A highly significant interaction emerged with factors WordType, PeriExtra, TempFront and Areas (*F*_2, 22_ = 14.012, *p* < 0.0002), revealing different CA circuits across the 12 area network between object- and action-related words. A main effect of Areas (*F*_2, 22_ = 265.721, *p* < 0.0001), indicating the different CA cell densities distributed across the network as noted above, namely higher CA cells in hubs than in secondary regions (*p* < 0.0001), and higher in secondary than in primary cortices (*p* < 0.0001). We separately ran a 3-way ANOVA on the data from the two systems, because of the significant interaction between peri- and extrasylvian areas. As expected, the extrasylvian system revealed a highly significant interaction of all 3 factors WordType, TempFront, and Areas (*F*_2, 22_ = 53.11, *p* < 0.0001), confirming the word category dissociation in the CA topographies and local cell-density distributions across the extrasylvian regions as suggested by Figures 2, 3. No significant differences between CA distributions of the 2 word types were found in the perisylvian areas (*F*_2, 22_ = 0.067, *p* = 0.93).

We further ran Bonferroni-corrected planned comparison tests (12 comparisons, corrected critical *p* < 0.0042) to investigate the differences between CA types that emerged after learning. Differences in CA-cell densities between word types and pairs of areas in the semantic systems were all significant (*p* < 0.0001), confirming the presence of a higher neuron-density in visual (V1, TO, and AT) than in motor (M1_L_, PM_L_, and PF_L_) areas for object-related words (*p* < 0.0001), and the opposite for action-related words (*p* < 0.0001). Analysis of the connector hubs (AT, PF_L_) also showed a significant difference between the 2 word types there, i.e. stronger action-related word CA cell densities in PF_L_ compared to AT (*p* < 0.0001), and the opposite for object-related words (*p* < 0.0001). As observed above, no significant differences emerged in the perisylvian areas (*p* = 0.029) between the word types. We further run the same statistical analysis on the object and action recognition data, which revealed similar results as the word production simulation, i.e., double dissociation between action and object-related words in the extrasylvian system (*F*_2, 22_ = 467.321, *p* < 0.0001) with no significant difference in perisylvian cortex (*F*_2, 22_ = 0.060, *p* < 0.91).

## Discussion

We investigated the neural mechanisms underlying word learning in a biologically constrained spiking model replicating connectivity and cortical features of the frontal, temporal and occipital areas to simulate aspects of semantic grounding in action and perception. The present neural-network showed

- Emergence of neuron circuits distributed across primary, secondary, and multimodal areas, as a result of simulating the grounding of word-forms in their semantically-related objects and actions (Figure 2). We call these “semantic circuits,” because they interlink articulatory-acoustic word-from information with referential semantic representations coded in motor and visual areas;
- Re-activation of the word-related circuits during word recognition exhibited the distinct consecutive neuronal and cognitive processes of word perception, word understanding and working memory (Figure 3);
- Higher neuron densities of the semantic circuits and prolonged activity in the multimodal areas, where all semantic and phonological information first converges;
- Pronounced semantic category-specificity primarily in the modality-preferential areas and moderate specificity also in multimodal areas for both word production and object and action recognition (Figures 4A,B).

The present simulations offer a neurobiological explanation of a wide range of recent experimental results about word meaning processing and make critical predictions about the functional role of multimodal-association hubs, secondary and primary cortical regions in language and semantic processing. Below, we provide a detailed discussion of the models and their results in light of previous empirical evidence, current semantic brain theories and its novel critical predictions.

### Semantic brain processes: data and models

Accumulating evidence emphasizes the relevance of several cortical regions for semantic processing, including inferior-frontal, superior- and anterior-temporal multimodal areas (Patterson et al., 2007; Binder et al., 2009; Pulvermüller, 2013), which are apparently relevant for all types of semantic processing, and modality-preferential areas, which seemingly take a category-specific role in semantics (Barsalou, 2008; Binder and Desai, 2011; Pulvermüller, 2013). Of great relevance in the current discussion about semantic grounding and “embodiment” is the contribution of modality-preferential areas including primary and secondary cortices, for example the motor and premotor cortex, or the primary and other “early” visual areas, in semantic processing. These areas, which had classically been seen as “perceptual” or “motor” in their function, seem to partake in and contribute to semantic processing, as a range of previous experimental studies showed. The present results fit the postulate of semantic grounding (Harnad, 1990) that, in order to know the meaning of a symbol, it is necessary to relate it to real world entities, for example, the word “grasp” to grasping actions and the word “house” to the typical visual shape of houses. Grounding in this sense needs to be implemented in semantic representations that reach into motor and sensory systems. Our simulations applying brain constrained modeling at different levels demonstrate grounding in this very sense, hence fitting (and explaining) the experimental results mentioned above.

Some attempts to integrate both category-general and category-specific semantic mechanisms into one theoretical framework have been proposed. The “hub-and-spoke” model postulates one single semantic hub in anterior-inferior-temporal lobe with category-specific spokes mainly in posterior brain areas (Ralph et al., 2017). This model explains crucial features of semantic dementia, but is inconsistent with hub-like properties of other multimodal areas (see Introduction) and, in addition, does not address the motor system's role in category-specific processing (Vukovic et al., 2017), along with some fine-grained differences in the ability to process specific semantic categories which result from different types of dementias (Shebani et al., 2017). Neurocomputational studies (Ueno et al., 2011; Chen et al., 2017) have investigated aspects of the hub-and-spoke model. However, as mentioned in the introduction, Chen et al. did not include all the brain areas for which experimental studies show a critical role in general semantic processing and they used learning mechanism (i.e., back-propagation—Ueno et al., 2011; Chen et al., 2017) which were criticized as implausible for cortical networks (Mazzoni et al., 1991; O'Reilly, 1998).

A claim about multiple semantic hubs has been made, in association with that about category-specific areas (Binder and Desai, 2011; Pulvermüller, 2013). However, formal neural-networks that could act as a foundation of a theory of semantic brain mechanisms did so far not reach the level of sophisticated neurobiologically constrained modeling with spiking neurons, realistic connectivity and learning. Earlier attempts were made using a preliminary version of the present architecture adopting non-spiking neurons (Garagnani and Pulvermüller, 2016; Tomasello et al., 2017). These previous models already suggest an explanation of category-general and category-specific semantic processing, but their conclusions were more limited by their less accurate modeling of neurophysiological and neuroanatomical features of the cortex.

### Novel contribution: increased brain-constraints

Here, we added important neurobiological constraints, introducing leaky integrate-and-fire neurons that transform their summed input non-linearly into discrete output in the form of spikes. Similarly to biological neurons, functional interaction within the present model was based on discrete spikes, whereas previous mean-field networks used continuous activity functions (i.e., graded-response neurons), a less realistic implementation. Using graded-response neurons makes it easier to build distributed neural circuits across multiple areas as a result of action-perception learning since this type of neuron retains an increased firing rate for more extended periods. It was, therefore, crucial to investigate the possibility of distributed circuit formation with spiking neurons, which show an activation (action potential) for a short moment and then go silent again.

Compared with earlier studies, the present network included a more realistic set of cortico-cortical fiber tracts, adding second-next area connections or “jumping links” (blue arrows Figures 1A,B) indicated by DTI/DWI studies. A recent neurocomputational study (Schomers et al., 2017) showed that these jumping links are instrumental for building verbal short-term memory, a capacity crucial for human language learning. Furthermore, previous exploratory implementation of “jumping links” in an extended semantic network of mean-field (non-spiking/gradually active) neuronal elements suggested a degree of over-activation in case of implementation of the rich set of cortico-cortical connections, thus preventing precise simulation of more realistic connectivity. The use of spiking neuronal cells, whose action potentials only last for 1 simulation time-step and therefore produced less activity overall compared with the graded-neuron network, opened the possibility to include additional connection pathways documented by recent research without running into over-activation problems. On the other hand, spiking-neuron networks with just next neighbor connections between areas (thus omitting the “jumping” links) ran into an under-activation problem, precisely because of the same feature (i.e., that spiking neurons lose their activity immediately). Thus, only the combined improvement of neuroanatomical (jumping connections) and neurophysiological (spiking) realism led to a functional network, which largely confirms conclusions formerly proposed on the basis of less realistic architectures. Incorporating significant biological detail into networks may be essential for obtaining a better understanding of the complex cortical mechanisms underlying semantic processing. Indeed, recent modeling results suggest that large-scale synchronous spiking within cell assembly circuits, also observed here, may be important for the binding of form to meaning during word learning and comprehension (Garagnani et al., 2017).

In summary, the comparison of less and more biologically constrained networks showed that improving the degree of realism does not always help. Moving from graded-response to spiking neurons alone renders an underactive network with little perspective on modeling semantic cognition, as the addition of a more detailed, elaborate and realistic connectivity structure on its own produces an overactive and thus, once again, dysfunctional networks. Only the parallel improvement on structural (anatomical) and functional (physiological) dimensions, that is, adding jumping links and spiking neurons, led to a functional network once again, which could confirm results from the earlier simulations obtained from the next-neighbor-connectivity and mean-field network, but provides a simulation at a more brain-constrained and therefore more realistic level.

### Emergence of distributed symbolic circuits

The present model imitates elementary processes of semantic learning, where word-forms are presented in the context of object (Vouloumanos and Werker, 2009) or action information (Tomasello and Kruger, 1992). In our model, the co-occurrence of objects or actions with word-forms was implemented as correlated neuronal activation patterns in the model's primary articulatory (M1_i_) and auditory (A1) along with either dorsolateral motor (M1_L_) or visual cortex (V1). The first significant finding of this study is that such information about the semantic grounding of symbols can be mapped reliably onto biologically constrained associative networks. Each pattern representing the pairing of one specific symbol and one specific action or object led to the formation of a distributed circuit of spiking neurons spread out across several areas of the architecture. Each of these distributed circuits acted as a coherent functional unit, with its interlinked neurons in sensory, motor and multimodal areas activating together. The formation of each circuit required the spreading of activity across the network and the selective strengthening of a significant number of partaking neurons. Such strengthening was substantial enough so that, after learning, “auditory input” was sufficient to revive the entire circuit, including its articulatory and semantic components. By comparing the mean-field next-neighbor model with the jumping-links spiking model, massive differences were revealed in the dynamics of cell assemblies activations during auditory word recognition (Figure 3). Whereas the mean-field model showed cascaded activation dynamics (with serial onset of activations and only partly overlapping activity of the hub areas AT, PF_L_), the full-fledged three-phase dynamics with perception (activation of auditory areas), ignition (near-simultaneous activation of cell assembly neurons dispersed across wide cortical areas), and working memory (reverberation of activity in part of the cell assembly) was only present in the spiking and fully connected model. Intriguingly, after ignition, activity retreats from modality-preferential areas (time step 12, Figure 3) to hub areas (time step 14), which predicts an “anterior shift” from visual and motor areas to adjacent-anterior connector hub regions in temporal and prefrontal cortex during working memory (see also Fuster, 2009; Pulvermüller and Garagnani, 2014; Pulvermüller, 2018).

Although the formation of each circuit was driven by correlated information in sensory and motor areas, widely distributed circuits with many neurons in multimodal convergence zones got active. The involvement of neurons in multimodal areas is explained by long-distance connectivity structure, in particular by the absence of direct long-distance connections between sensory and motor areas; to bind information across modalities, activity must travel through connector hub areas (also called convergence zones, Damasio, 1989) bridging between sensorimotor cortices. It is important to emphasize, however, that while the presence of connector hubs in the model is a (neuroanatomically motivated) structural feature, the result that the learned action and object word circuits reach *both* extrasylvian connector hubs AT and PF_L_–hence forming semantic hubs—is not trivial, and could not be *a priori* predicted^1^. In other words, while the presence of *connector* hubs is a structural feature of the model, the formation of *semantic hubs* is not, and constitutes one of its crucial emergent properties.

The spontaneous formation of internal semantic circuits spanning the entire spiking neural network is a direct consequence of neurobiological principles modeled in the architecture that are known to govern the human brain. As discussed below, the activation of the learned distributed circuits explains relevant “semantic area activations” seen in neuroimaging experiments (for further discussion, see Garagnani and Pulvermüller, 2013; Tomasello et al., 2017).

### Explaining multiple semantic hubs

Not only did our model firmly bind neurons in multimodal areas to sensorimotor neurons involved in semantic processing, but, within each circuit, the proportion of these multimodal-area neurons was even greater than the percentage of circuit neurons in primary and secondary areas. On first view, this appears as surprising, because, during pattern presentation, sensory and motor neurons were directly stimulated together, whereas multimodal areas were activated only indirectly, by activity spreading from primary areas. However, the multimodal areas occupy a central location in the network topology because they bridge between sensory and motor areas, and therefore receive near-simultaneous convergent input from different (here, three) systems during learning. Such convergence also takes advantage of the higher “degree” of connectivity characterizing multimodal areas and of their resultant role as “connector hubs,” for which a special role in cognition has previously been proposed (van den Heuvel and Sporns, 2013). The cumulative effect of correlated inputs through several pathways converging on multimodal hubs accounts for their higher neuron-densities and their resultant major contribution to semantic circuit function. Thus, given that large fractions of the neurons of all semantic circuits were located in connector hubs, the model explains the prominent role of these connector regions in general semantic processing, which is due to both, the well-known pre-existing neuroanatomical connectivity and the correlated neuronal activity during word learning.

Crucially, the model implicates and explains not only one, but at least four experimentally observed “semantic hub” areas. One of these is in anterior-temporal lobe, providing a theoretical foundation for the critical postulate of the hub-and-spoke model (Patterson et al., 2007). Other semantic hubs are in superior-temporal-parabelt and in inferior- and dorsolateral-prefrontal cortex, where other models postulate sites of general semantic processing (Posner and Pavese, 1998; Bookheimer, 2002; Tate et al., 2014; Schomers and Pulvermüller, 2016; Carota et al., 2017). Our model, therefore, fits (and explains) data indicating the presence of frontal and temporal semantic hub areas, thus reconciling extant experimental evidence for a range of regions generally involved in conceptual processing (for reviews, see Kiefer and Pulvermüller, 2012; Pulvermüller, 2013).

### Explaining category-specificity

We modeled the learning and processing of two different semantic categories: object- and action-related words. The formation of semantic circuits was driven by sensorimotor pattern information, involving visual cortex activity for object words and hand-motor cortex activity for action words. The respective other input system was activated with random noise to model the variable action output (visual input) in the context of specific visual objects (actions). Such uncorrelated noisy activity counters the spontaneous extension of neuron circuits toward inactive areas (Doursat and Bienenstock, 2006). Notably, as a consequence of the differential sensorimotor activation patterns, different circuit topographies developed across the areas for both word production and action or object recognition: circuits storing action-related information reached into the motor cortices (M1_L_-PM_L_) but not or less into visual areas (V1-TO), and vice versa for object words. Semantic circuits with different cortical topographies, which are a result of correlated neuronal activity in different sensorimotor areas during language learning, can therefore explain the emergence of category-specific semantic contributions of different cortical areas.

We take this observation as a proof-of-concept that the present type of spiking and jumping network is capable of spontaneously developing semantic-category specificity replicating a number of studies revealing neuroimaging and neuropsychological dissociations between action verbs and object nouns or between nouns sub-categories related to animals and tools (Damasio and Tranel, 1993; Martin et al., 1996; Martin, 2007; Moseley and Pulvermüller, 2014; Kemmerer, 2015). Interestingly, some category specificity was revealed in the semantic hubs, although it was less pronounced compared with primary and secondary areas. This area category-specific activation predicted by the model (Figure 4) seems to be of graded nature, with stronger category effect in the primary areas than in secondary areas and stronger in the secondary than in the hub areas and awaits experimental validations. The moderate category specificity predicted in the semantic hub areas is in line with recent evidence that semantic dementia patients due to anterior-temporal lesion show category-specific semantic impairments (Pulvermüller et al., 2010; Gainotti, 2012; Shebani et al., 2017), which sits less well with the suggested general-semantic function across all semantic types (Patterson et al., 2007).

It needs to be emphasized that most previous studies on semantics have investigated action and object words taken from natural languages, focusing mostly on the noun-verb distinction, which makes it difficult to control for all psycholinguistic proprieties and especially, when these words were acquired (e.g., Moseley and Pulvermüller, 2014). If we take our present simulations as models of concrete action verb vs. object noun processing, there is a good fit with the data, as these semantically and lexically different word types tend to differentially activate motor regions or ventral visual areas respectively (Damasio et al., 1996; Martin et al., 1996; Pulvermüller et al., 1999, 2014; Vigliocco et al., 2004; Martin, 2007; Moseley et al., 2013). However, note that the “action” and “object words” simulated here capture the differential action- and object-relatedness of many verbs and nouns, but not the lack of such semantic differences seen between abstract verbs/nouns and certainly not the combinatorial, or distributional differences between word categories, which result from their differential placements in specific grammatical contexts. Hence, for directly comparing the predictions of the present simulations to empirical data, it will be advantageous to perform analogous learning experiments and brain imaging studies to investigate *where* in the brain the neural signatures of novel object and action words first emerge. Nevertheless, the present simulation demonstrate the validity of a neurobiological theory of language processing (see Introduction, and Damasio, 1989; Pulvermüller, 2013), in which the mutual interaction of a set of neurobiological principles at work within anatomically-realistic structures and Hebbian learning are sufficient for explaining the emergence of semantic hubs and category specificity in the human brain.

It may be worthwhile to point to additional limitations of the present work along with possible extensons in the future. When an infant learns a new action word (e.g., “grasp”), by hearing a novel word form while performing the related action toward an object, concurrent activity might be present not just in the perisylvian language areas and motor cortices, but also in the visual occipital-parietal “where” stream (Mishkin and Ungerleider, 1982; Mishkin et al., 1983), which was not implemented here. Therefore, an important extension of the present model would be to include parietal areas and the dorsal visual-where stream. Inclusion of left parietal areas would also be strongly motivated experimentally, as they are well known to play a role in general language processing (Pulvermüller and Fadiga, 2010) and also in category-specific processing of prepositions, number and tool words (Dehaene, 1995; Binder and Desai, 2011; Tschentscher et al., 2012; Shebani et al., 2017). Further model extensions should address other forms of language learning. Here we investigate but one aspect of word meaning acquisition, namely associative learning between a word and its referents, which represents only a very basic step of semantic learning. To capture other types of semantic learning, the emergence of semantic knowledge from variable contexts needs to be covered along with the semantic grounding of words learned from texts, where semantic links may be explained by co-activation of linguistic representations. Future work may address with realistic neuronal networks how, based on a kernel of early acquired words semantically grounded in referent object and action contexts, the co-occurrence of words in texts can lead to the formation of novel semantic circuits and semantic representations (Harnad, 2011; Stramandinoli et al., 2012). Furthermore, future simulations should extend the present work by investigating how combinatorial grammatical binding between pre-learnt and whole-form-stored lexical units emerges from correlated activity in co-activated neuronal circuits (see Pulvermüller, 2010).

Still, already in its current form, the present computational model makes critical predictions (some of which we spelled out in detail in discussion above) about how meaning is acquired, processed and stored in the human brain. Compared with earlier similar work, the spiking-and-jumping neural network developed in this work is based on a wider range of biological principles and features of the human brain, such as neurophysiological dynamics of spiking pyramidal cells, synaptic modification by way of Hebbian learning, local lateral inhibition and area-specific global regulation mechanisms, uncorrelated white noise present in all neurons during learning, brain-like connectivity structure based on neuroanatomical evidence. Therefore, the present model provides a sophisticated mechanistic explanation of the differential involvement of semantic cortical regions.

## Conclusion

We used a biologically constrained neurocomputational model mimicking cortical features and connectivity of frontal, temporal and occipital cortices to simulate the brain mechanisms of word meaning acquisition. Extending our earlier work (Garagnani and Pulvermüller, 2016; Tomasello et al., 2017) by introducing, for the first time, spiking neuronal cells in a neuroanatomical constrained model with brain like connectivity, we show that Hebbian associative learning and connectivity together are sufficient to account for the emergence of general semantic areas (“semantic hubs”), as well as specific contributions of others modality-preferential ones to the processing of specific semantic categories. The present simulation results show that neurobiologically constrained networks can fruitfully contribute to bridging the gap between cellular-level mechanisms, behavior and cognition by integrating brain theory with experimental data.

## Author contributions

RT conceived the study, conducted the experiments, analyzed the data, and wrote the paper. MG, TW, and FP supervised the study and contributed to paper writing.

### Conflict of interest statement

The authors declare that the research was conducted in the absence of any commercial or financial relationships that could be construed as a potential conflict of interest.

## Footnotes

^1^Note that the linkage of a perisylvian word circuit with semantic information coming from the visual (or motor) system does not necessarily have to go through connector hub PF_L_ (or AT).
